# The Shape-Shifting Heart: Recurrent Takotsubo Cardiomyopathy Manifesting as Two Variants in One Patient

**DOI:** 10.7759/cureus.102380

**Published:** 2026-01-27

**Authors:** Sukhila Reddy, Muhammad Asif, Mounica Vorla, Muhammad Hasib Khalil

**Affiliations:** 1 Cardiology, Carle Foundation Hospital, Urbana, USA; 2 Internal Medicine, Carle Foundation Hospital, Urbana, USA

**Keywords:** apical variant, mid-ventricular variant, stress-induced cardiomyopathy, takotsubo cardiomyopathy, transient left ventricular dysfunction

## Abstract

Takotsubo cardiomyopathy (TCM), also known as stress-induced cardiomyopathy, is a transient left ventricular dysfunction that mimics acute coronary syndrome but typically resolves within weeks. Its recurrence is uncommon, and recurrence with distinct morphologic variants is exceedingly rare.

We report a 75-year-old female with peripheral arterial disease, paroxysmal atrial fibrillation, sick sinus syndrome status post pacemaker, and prior apical variant TCM in 2022. She underwent a right lower extremity surgery. Two days postoperatively, she developed acute chest pain and troponin elevation. Echocardiography revealed new left ventricular dysfunction with an ejection fraction (EF) of 35-40% and hypokinesis of the anteroseptal and anterior walls. Coronary angiography demonstrated mild nonobstructive coronary artery disease (CAD). Ventriculography confirmed the mid-ventricular variant of TCM. This contrasted with her prior episode in 2022, when she developed apical ballooning variant TCM with an EF of 25-30% during admission for *Escherichia coli* bacteremia. In both episodes, her EF normalized on follow-up imaging.

This case highlights the protean nature of TCM and the rare phenomenon of recurrence with different morphologic variants. Recognition of variant-specific triggers and long-term surveillance are essential in patients with prior episodes.

## Introduction

Takotsubo cardiomyopathy (TCM), also known as stress-induced cardiomyopathy, is a syndrome characterized by transient left ventricular dysfunction that mimics acute coronary syndrome but in the absence of angiographic evidence of obstructive coronary artery disease (CAD) or acute plaque rupture that typically resolves within weeks. It was first described in Japan in 1990, named after the “octopus trap” shape of the left ventricle during systole [1,2]. It accounts for 1-2% of suspected acute coronary syndromes [2]. While recurrence occurs in 1-6% of patients [3], the vast majority of reported recurrences involve the same morphologic pattern, and recurrence with different phenotypic variants is exceedingly rare [4,5]. Variants include apical, mid-ventricular, basal, and focal types [6].

As a result, the mechanisms underlying regional myocardial susceptibility and variant switching within the same patient remain poorly understood, representing an important gap in the current literature.

This phenomenon challenges current understanding of catecholamine-mediated myocardial stunning, microvascular dysfunction, and regional myocardial susceptibility. Cases demonstrating recurrent TCM with distinct morphologic variants provide a unique clinical model to explore these mechanisms and their relationship to differing physical and systemic stressors. We present a case of recurrent TCM manifesting as two distinct morphologic variants, underscoring the dynamic vulnerability of myocardial segments under varying stressors.

The objectives of this report are to highlight the potential for recurrent TCM to present with different morphologic patterns within the same patient, an infrequently described and clinically relevant phenomenon, to explore the role of variant-specific triggers in influencing clinical presentation and severity, to emphasize the importance of coronary angiography and ventriculography in differentiating TCM from ischemic heart disease, and to underscore the need for long-term clinical surveillance in patients with prior TCM episodes due to the risk of recurrence.

## Case presentation

A 75-year-old female with peripheral arterial disease, paroxysmal atrial fibrillation, sick sinus syndrome status post pacemaker, pulmonary hypertension, and prior apical variant TCM in 2022 was admitted for critical limb ischemia and nonhealing wounds of the right leg. She underwent a right superficial femoral artery-posterior tibial bypass using an ipsilateral reversed great saphenous vein graft without complications.

**Figure 1:**
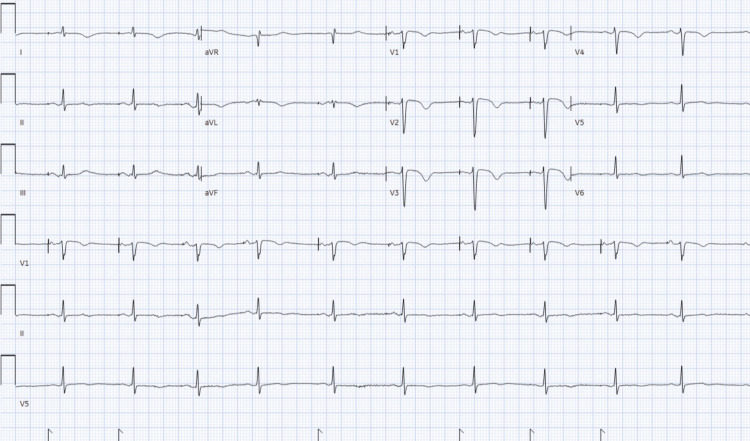
Electrocardiogram showing atrial paced rhythm with nonspecific T-wave inversions.

Two days later, she developed acute retrosternal pressure-like chest pain (8/10) with diaphoresis and dyspnea. The electrocardiogram (ECG) showed an atrial paced rhythm with nonspecific T-wave inversions (Figure 1).

**Figure 2:**
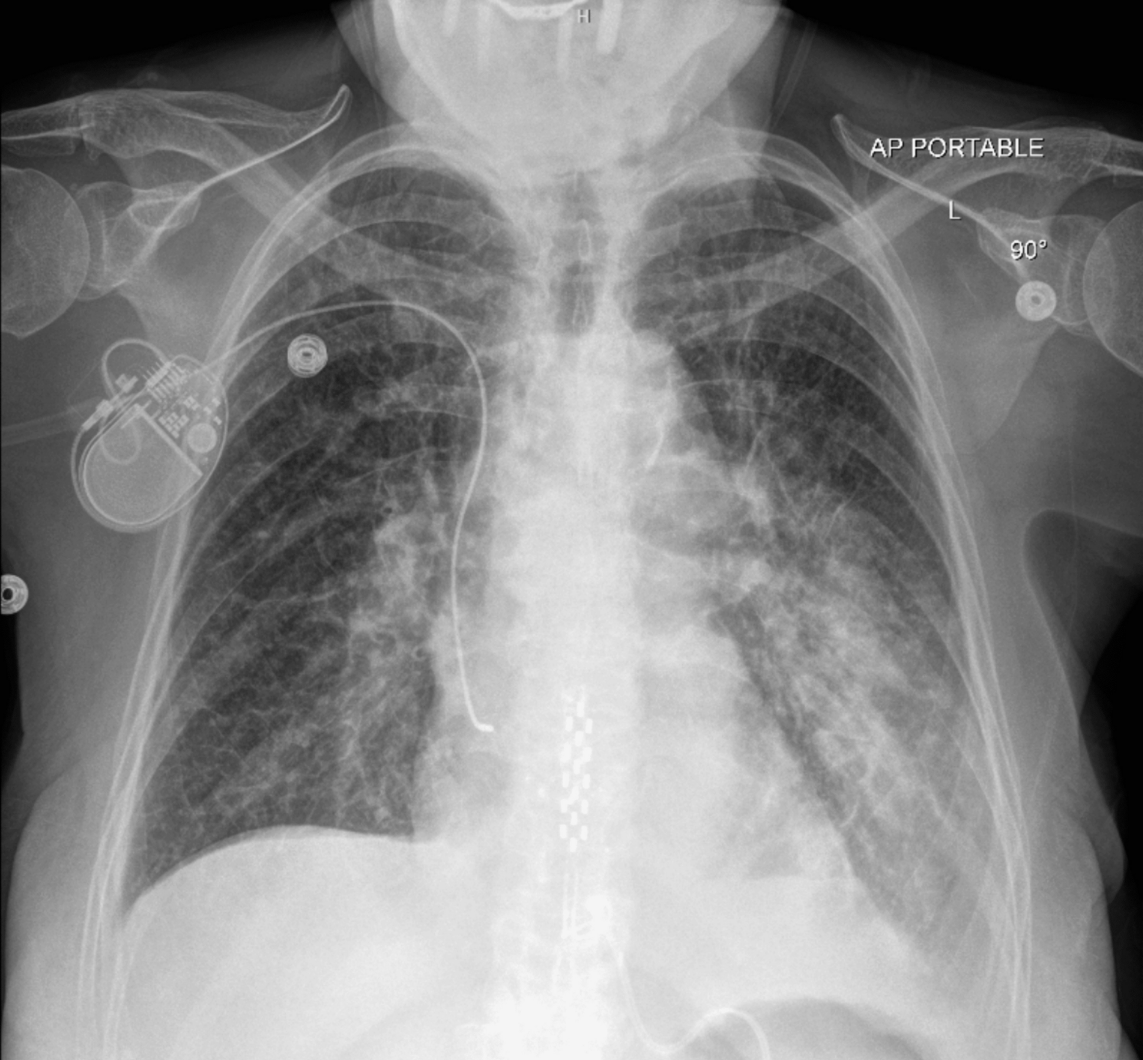
Chest X-ray demonstrating diffuse interstitial markings consistent with pulmonary edema.

High sensitivity troponin peaked at 2287 ng/L, and B-type natriuretic peptide (BNP) was 736 pg/mL. She was diagnosed with acute coronary syndrome and started on intravenous heparin, aspirin, and clopidogrel. Chest X-ray demonstrated diffuse interstitial markings consistent with pulmonary edema (Figure 2).

Diuretics were initiated; however, she developed hypotension requiring ICU transfer and vasopressor support.

**Figure 3:**
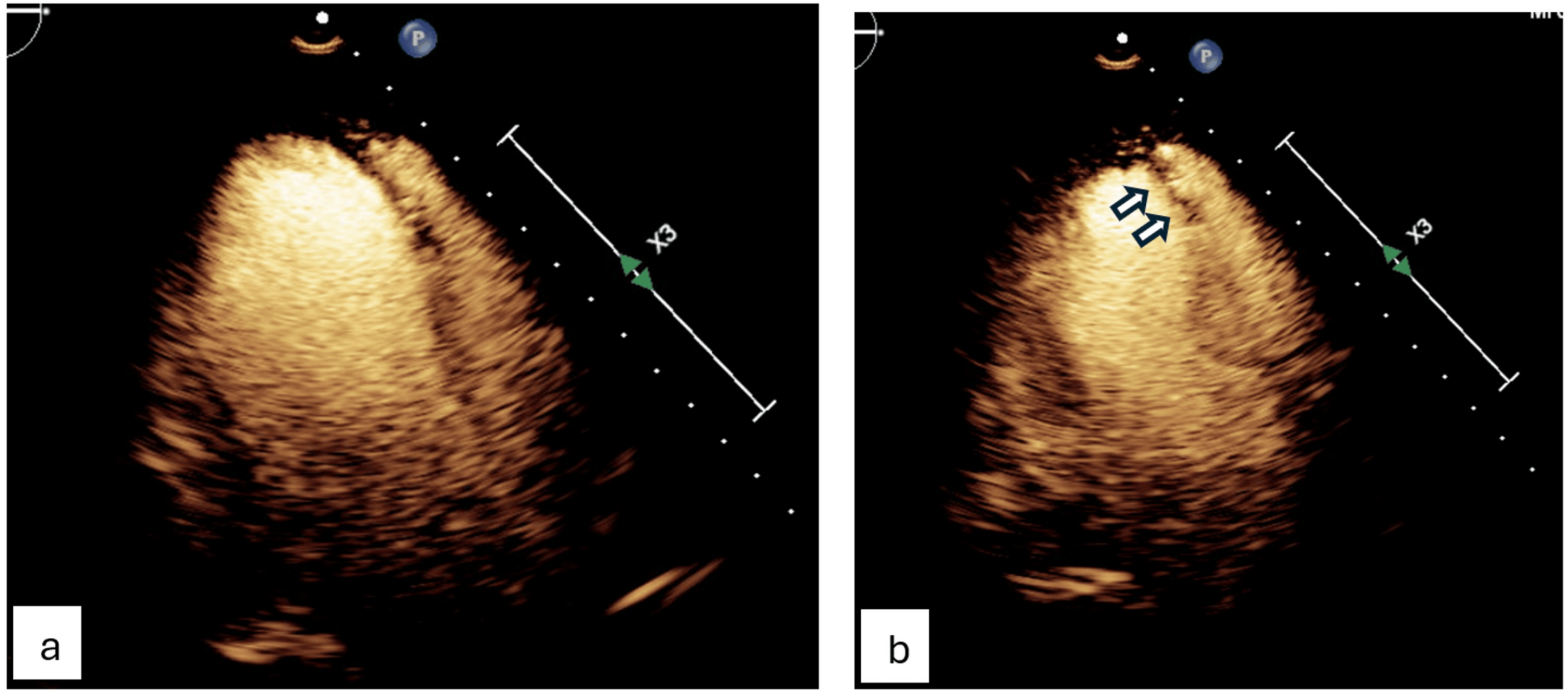
Transthoracic echocardiogram (parasternal long-axis view) demonstrating anteroseptal hypokinesis. (a) Wall motion in diastole. (b) Wall motion in systole.

**Figure 4:**
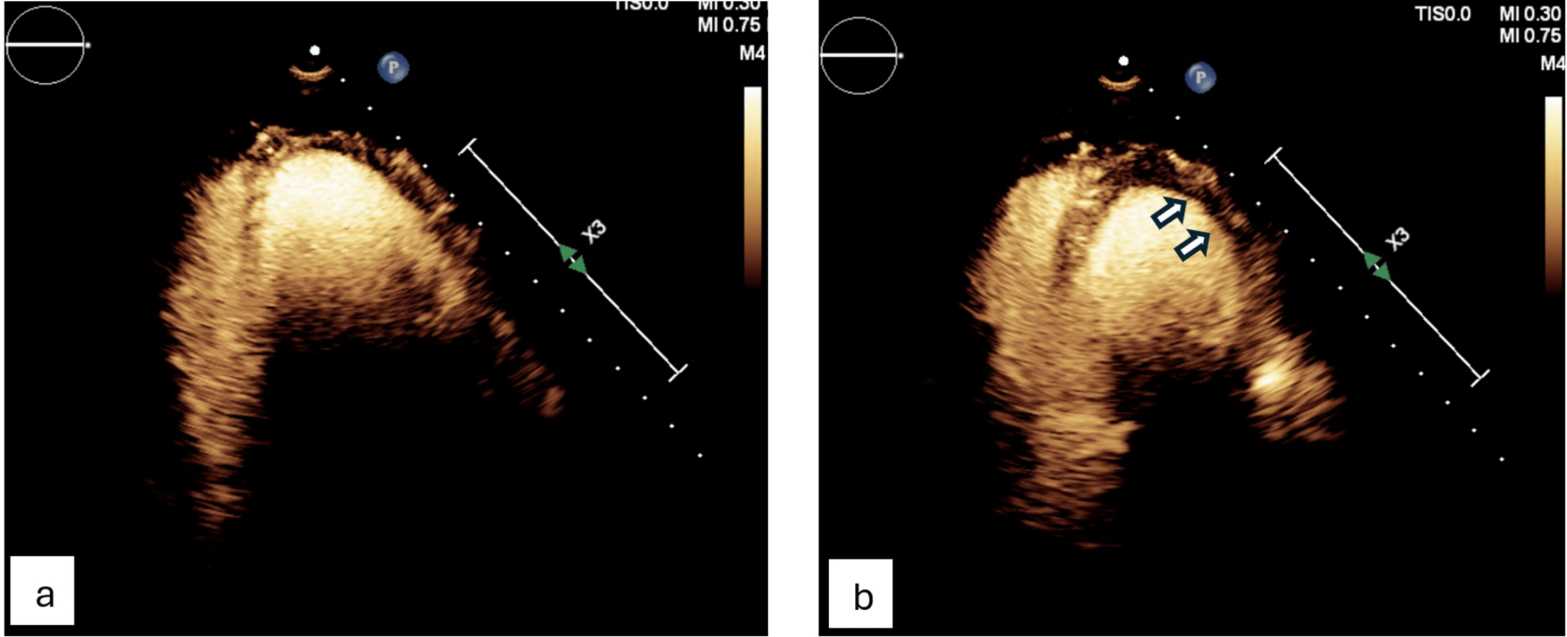
Transthoracic echocardiogram (apical two-chamber view) showing hypokinesis of the anterior wall. (a) Wall motion in diastole. (b) Wall motion in systole.

Transthoracic echocardiogram (TTE) revealed new left ventricular dysfunction with an ejection fraction (EF) of 35-40% and hypokinesis of the anteroseptal and anterior walls (Figures 3, 4).

**Figure 5:**
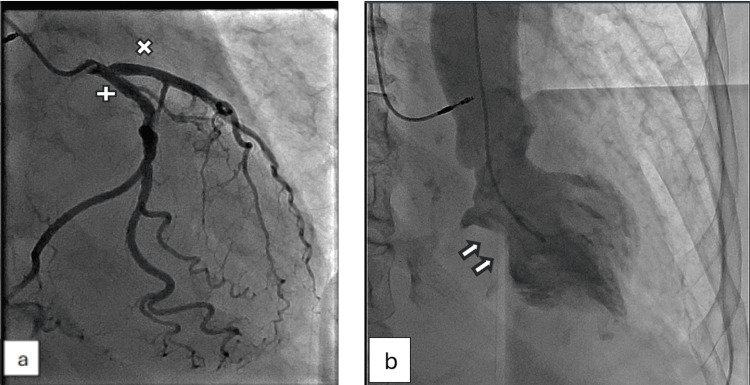
Coronary angiography revealing mild non-obstructive coronary artery disease. (a) Left anterior descending artery (x) and left circumflex artery (+). (b) Right coronary artery.

Coronary angiography showed mild non-obstructive CAD (Figure 5).

**Figure 6:**
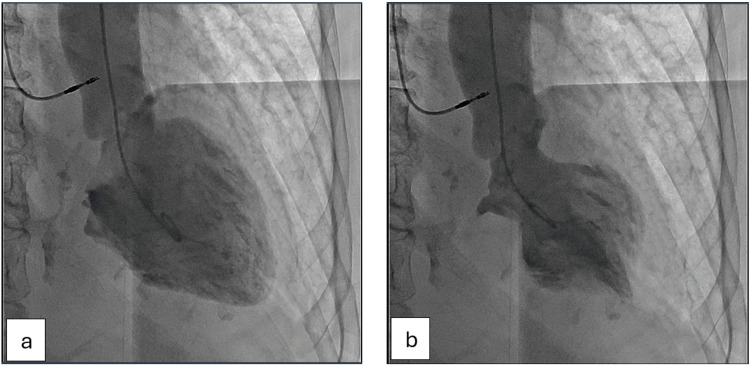
Left ventriculography showing mid-ventricular ballooning variant of takotsubo cardiomyopathy. (a) In diastole. (b) In systole.

Left ventriculography confirmed mid-ventricular variant TCM (Figure 6).

Her respiratory status improved, diuretics were discontinued, and she was discharged on maximum tolerated guideline-directed medical therapy (GDMT). A follow-up echocardiogram done nine months later demonstrated normal EF.

**Figure 7:**
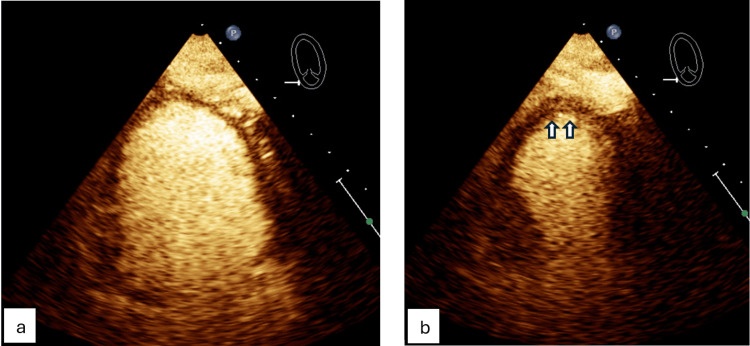
Prior transthoracic echocardiogram (apical four-chamber view) showing apical ballooning of the left ventricle. (a) Wall motion in diastole. (b) Wall motion in systole.

In 2022, during hospitalization for *Escherichia coli* bacteremia, she developed chest pain with troponin elevation. TTE revealed apical ballooning of the left ventricle with an EF of 25-30% (Figure 7).

Coronary angiography showed mild nonobstructive CAD. She was diagnosed with apical variant TCM and discharged on GDMT. Follow-up echocardiogram demonstrated normalization of EF.

## Discussion

TCM is thought to result from catecholamine-mediated myocardial stunning. Plasma catecholamine levels in TCM are markedly elevated compared with those in acute myocardial infarction [5]. Excess catecholamines may cause direct myocyte injury, coronary microvascular spasm, or dynamic left ventricular outflow obstruction [6].

Proposed mechanisms underlying this condition include a catecholamine surge with heterogeneous β-adrenergic receptor distribution across left ventricular segments, which may explain why different stressors result in distinct morphologic variants [7]. In addition, coronary microvascular dysfunction, characterized by microvascular spasm and impaired endothelial function, may lead to transient myocardial ischemia in the absence of obstructive CAD [8]. Autonomic imbalance marked by heightened sympathetic activity and reduced parasympathetic tone further predisposes individuals to exaggerated stress responses [9]. The disproportionate involvement of postmenopausal women suggests a protective role of estrogen against catecholamine-mediated myocardial toxicity [10]. Lastly, genetic susceptibility may contribute, as familial clustering has been described, although no definitive causative gene has yet been identified [11].

From a clinical standpoint, TCM should be suspected when chest pain and biomarker elevation occur in the setting of diffuse or atypical wall-motion abnormalities. Modest troponin rise relative to ventricular dysfunction, and rapid reversibility on follow-up imaging are features that help distinguish it from true ischemic myocardial injury [1,2,7].

Recurrence of TCM has been reported in 1-6% of patients [3,12]. Most recurrences involve the same variant, typically apical ballooning [13]. Rare cases demonstrate recurrence with different variants, as seen in our patient [4,14,15]. Singh et al. described patients with recurrent TCM manifesting initially as apical ballooning, followed by mid-ventricular or basal variants [4]. Shaw et al. reported that recurrence may involve either identical or distinct morphologic patterns, suggesting dynamic myocardial susceptibility [3]. This case is unique not only due to recurrence but also due to the manifestation of two distinct morphologic variants triggered by different stressors.

The mid-ventricular variant is particularly associated with physical stressors such as surgery, anemia, or shock, and may carry worse hemodynamic compromise [16]. In contrast, the apical variant is more often linked to emotional stress or systemic illness (Table 1) [17].

**Table 1:** Comparison of apical versus mid‑ventricular takotsubo cardiomyopathy. Source: Akashi et al. (2003) [6], Singh et al. (2014) [4], Shaw et al. (2023) [3], Ghadri et al. (2018) [15], Eitel et al. (2011) [16], and Bybee et al. (2004) [17].

Feature	Apical variant	Mid-ventricular variant
Typical trigger	Emotional stress, systemic illness [6,17]	Physical stress (surgery, anemia, shock) [15,16]
Wall motion pattern	Apical ballooning with basal sparing [6,16]	Hypokinesis of mid-left ventricle segments, apical/basal sparing [4,15,16]
Ejection fraction	Often severely reduced (20–30%) [6,16]	Moderately reduced (30–40%) [4,15]
Clinical presentation	Chest pain, dyspnea, mimics acute coronary syndrome [6,17]	More frequent hemodynamic compromise, pulmonary edema [15,16]
Prognosis	Generally favorable, recurrence rare [3,12,13]	May carry a higher risk of complications [15,16]

This distinction aligns with our patient's presentations, with postoperative stress precipitating the mid-ventricular variant and systemic infection precipitating the apical variant.

This case underscores the diagnostic challenge: troponin elevation, wall motion abnormalities, and chest pain mimic acute coronary syndrome. Coronary angiography and ventriculography remain essential to exclude obstructive CAD and confirm variant morphology.

Management is largely supportive and includes β-blockers and angiotensin-converting enzyme inhibitors or angiotensin receptor blockers during the acute and recovery phases, although evidence for prevention of recurrence remains limited [5,12,15]. Follow-up echocardiography is recommended to confirm recovery of left ventricular function, typically within weeks to months after the acute event [5,15].

Long-term prognosis is generally favorable, but recurrence risk persists. Patients with recurrent TCM may have a higher risk of adverse outcomes, warranting close follow-up [18,19].

Learning points

TCM can recur with different morphologic variants in the same patient. Variant-specific triggers may influence clinical presentation and severity. Coronary angiography and ventriculography remain essential to distinguish TCM from ischemic heart disease. Long-term surveillance is warranted in patients with prior TCM episodes due to recurrence risk.

## Conclusions

Recurrent TCM with different morphologic variants is rare. This case illustrates the protean nature of stress cardiomyopathy, the importance of distinguishing it from ischemic heart disease, and the need for long-term surveillance in patients with prior episodes.

Future studies are warranted to better elucidate the mechanisms underlying variant-specific myocardial involvement. Prospective investigations aimed at identifying predictors of recurrence will be beneficial.
